# Platelet-derived factors impair placental chorionic gonadotropin beta-subunit synthesis

**DOI:** 10.1007/s00109-019-01866-x

**Published:** 2019-12-20

**Authors:** Désirée Forstner, Sabine Maninger, Olivia Nonn, Jacqueline Guettler, Gerit Moser, Gerd Leitinger, Elisabeth Pritz, Dirk Strunk, Katharina Schallmoser, Gunther Marsche, Akos Heinemann, Berthold Huppertz, Martin Gauster

**Affiliations:** 1 Division of Cell Biology, Histology and Embryology, Gottfried Schatz Research Center, Medical University of Graz, Neue Stiftingtalstraße 6, II, 8010 Graz, Austria; 2 Cell Therapy Institute, Spinal Cord Injury and Tissue Regeneration Center Salzburg (SCI-TReCS), Paracelsus Medical University, Salzburg, Austria; 3 Department of Transfusion Medicine and Spinal Cord Injury and Tissue Regeneration Center Salzburg (SCI-TReCS), Paracelsus Medical University, Salzburg, Austria; 4 Division of Pharmacology, Otto Loewi Research Center, Medical University of Graz, Graz, Austria

**Keywords:** Pregnancy, Placenta, Development, Platelets, Human chorionic gonadotropin

## Abstract

**Abstract:**

During histiotrophic nutrition of the embryo, maternal platelets may be the first circulating maternal cells that find their way into the placental intervillous space through narrow intertrophoblastic gaps within the plugs of spiral arteries. Activation of platelets at the maternal-fetal interface can influence trophoblast behavior and has been implicated in serious pregnancy pathologies. Here, we show that platelet-derived factors impaired expression and secretion of the human chorionic gonadotropin beta-subunit (βhCG) in human first trimester placental explants and the trophoblast cell line BeWo. Impaired βhCG synthesis was not the consequence of hampered morphological differentiation, as assessed by analysis of differentiation-associated genes and electron microscopy. Platelet-derived factors did not affect intracellular cAMP levels and phosphorylation of CREB, but activated Smad3 and its downstream-target plasminogen activator inhibitor (PAI)-1 in forskolin-induced BeWo cell differentiation. While TGF-β type I receptor inhibitor SB431542 did not restore impaired βhCG production in response to platelet-derived factors, Smad3 inhibitor SIS3 interfered with CREB activation, suggesting an interaction of cAMP/CREB and Smad3 signaling. Sequestration of transcription co-activators CBP/p300, known to bind both CREB and Smad3, may limit βhCG production, since CBP/p300 inhibitor C646 significantly restricted its forskolin-induced upregulation. In conclusion, our study suggests that degranulation of maternal platelets at the early maternal-fetal interface can impair placental βhCG production, without substantially affecting morphological and biochemical differentiation of villous trophoblasts.

**Key messages:**

- Maternal platelets can be detected on the surface of the placental villi and in intercellular gaps of trophoblast cell columns from gestational week 5 onwards.
- Platelet-derived factors impair hCG synthesis in human first trimester placenta.
- Platelet-derived factors activate Smad3 in trophoblasts.
- Smad3 inhibitor SIS3 interferes with forskolin-induced CREB signaling.
- Sequestration of CBP/p300 by activated Smad3 may limit placental hCG production.

**Electronic supplementary material:**

The online version of this article (10.1007/s00109-019-01866-x) contains supplementary material, which is available to authorized users.

## Introduction

Successful human pregnancy is initiated by implantation of the blastocyst into the decidua, i.e. the highly differentiated endometrium, which provides the ground for subsequent placentation. At this early stage of pregnancy, a sequence of complex and tightly regulated processes guarantees the development of the placenta, which functions as an immunological barrier and allows the supply of maternal nutrients, as well as the exchange of respiratory gases and the synthesis of endocrine factors to adapt the maternal physiology to the growing embryo. One key event in human placentation is the establishment of the uteroplacental circulation, enabling direct contact of maternal blood with placental chorionic villi. Placental extravillous trophoblasts invade the maternal decidua, where they accumulate and form cellular plugs that obstruct maternal arterial blood flow to the developing placental villous tissue until the end of the first trimester of pregnancy. However, presence of loosely cohesive trophoblast plugs with clear capillary-sized channels with flow toward the intervillous space has been suggested to enable initial microvascular flux by 7 weeks of gestation [1]. These channels seem to be the first signs of subsequent plug disintegration and complete remodeling of maternal spiral arteries into wide-bore, low-resistance conduits.

The time when trophoblast plugs become loosely cohesive can be considered the time when platelets—as the first circulating maternal cells—find their way through the narrow intercellular gaps into the intervillous space. Previous immunostaining of early human placental tissues detected platelets in maternal spiral arteries, attaching to the surface of invaded trophoblasts or to vessel walls that were infiltrated by perivascular trophoblasts [2]. In the same study, in vitro experiments showed that CD41^+^ platelets adhered to isolated CD146^+^ extravillous trophoblasts and that most of the platelets expressed P-selectin on their cell surface, suggesting that they had been activated. Moreover, co-culture with platelets enhanced invasion of trophoblasts and morphological observations suggested that platelet-derived factors induced extravillous trophoblast differentiation toward an endovascular phenotype [2]. We have recently shown maternal platelets on villous explant cultures from human first trimester placenta, indicating that adherence of maternal platelets to the villous surface is a common process even in early stages of human pregnancy [3]. However, later on in pregnancy, exaggerated activation of aggregated platelets at the maternal-fetal interface is implicated in serious pregnancy pathologies [4]. Accordingly, procoagulant platelet- or endothelial-derived extracellular vesicles have been suggested to trigger accumulation of activated platelets in the murine placenta, causing inflammasome activation in trophoblasts and leading to characteristic hallmarks of the pregnancy pathology preeclampsia [5].

Interestingly, initiation of microvascular flux through capillary-sized channels of loosely cohesive trophoblast plugs during the second half of the first trimester of pregnancy coincides with a steep rise in placental secretion of the pregnancy hormone human chorionic gonadotropin (hCG). In human pregnancy, hCG levels rise exponentially during the first 7 weeks, to peak at 10 weeks of gestation and decline slowly until term [6]. The major function of hCG during human pregnancy is driving hemochorial placentation, including regulation of uterine, fetal, and placental growth, as well as protecting pregnancy from myometrial contraction and from immune rejection [6]. In terms of placental growth, hCG has been shown to trigger differentiation and fusion of villous trophoblasts with the overlying, so-called syncytiotrophoblast, which builds the epithelial-like surface of placental chorionic villi [7]. HCG is predominantly synthesized in the syncytiotrophoblast, and thus acts in a paracrine/autocrine way on villous growth, which is mainly driven by trophoblast growth at this early stage in pregnancy. The temporal overlap of the moment when maternal platelets may get first contact with placental chorionic villi and the steep rise in hCG tempted us to test the hypothesis whether or not platelet-derived factors play a regulatory role in the differentiation of the villous trophoblast and its production of hCG.

## Results

### Maternal platelets can be detected at the early maternal-fetal interface

Immunohistochemistry of 31 human first trimester placenta tissues for platelet marker CD42b detected maternal platelets on the surface of placental villi from gestational week 5 up to week 12. Maternal platelets were either detected on the apical surface of the syncytiotrophoblast (Fig. 1a, b) or on initial perivillous fibrinoid deposits (Fig. 1c). Interestingly, some cases showed platelets between the villous cytotrophoblast and the syncytiotrophoblast layer (Fig. 1d). Staining of serial sections for HLA-G (Fig. 1e), a marker for the invasive extravillous trophoblast and CD42b (Fig. 1f), detected maternal platelets in intercellular spaces of HLA-G-positive trophoblasts in anchoring parts of cell columns, which attach placental villi to the maternal decidua. Moreover, staining of adjacent first trimester placental tissue sections for CD42b as well as for HLA-G and von Willebrand factor (vWF), as a marker for endothelial cells, showed maternal platelets accumulating in close proximity of fragmentary trophoblast plugs in uterine blood vessels (Fig. 1g, h). Overall, the immunohistochemical survey revealed that 29 out of 31 (93.6%) first trimester placenta cases showed platelets on the surface of placental villi (Suppl. Table 1). Of note, the occurrence of platelets adhering on initial fibrinoid deposits increased with gestational age.

**Figure:**
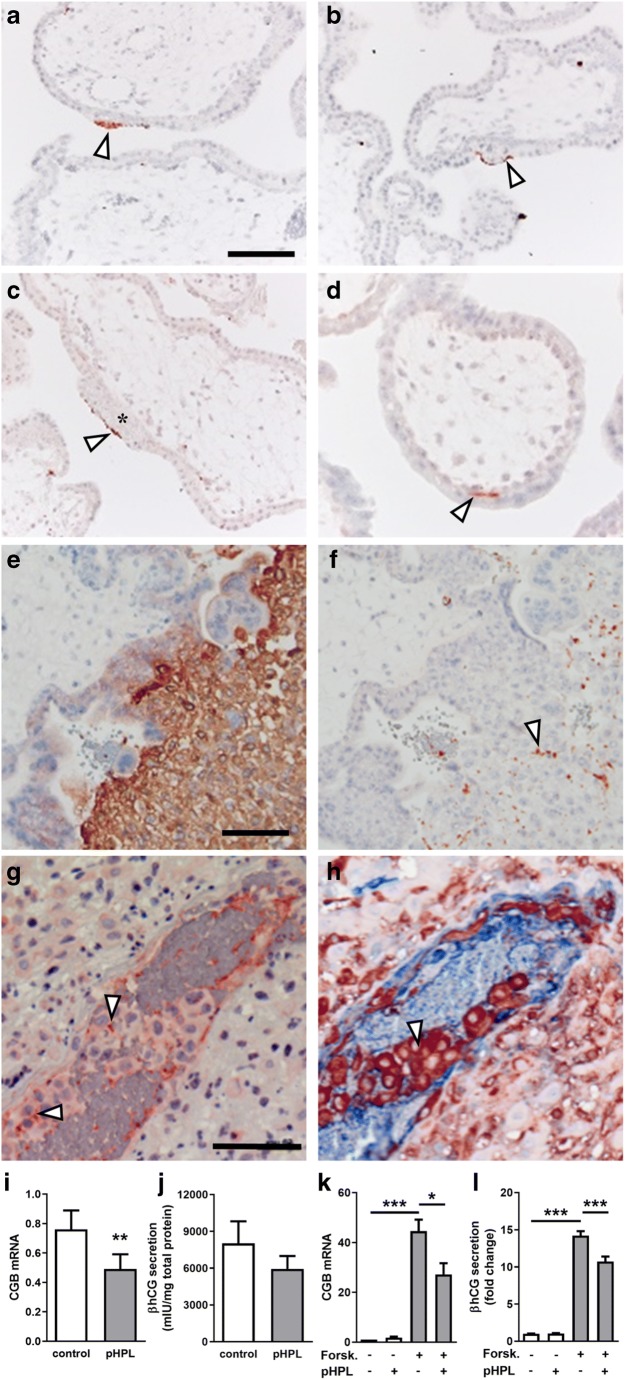

### Platelet-derived factors reduce βhCG synthesis in the villous trophoblast

In order to elucidate the consequence of putative platelet degranulation at the early maternal-fetal interface, placental explants and the trophoblast cell line BeWo were incubated in the presence or absence of pooled human platelet lysate (pHPL). Presence of pHPL significantly decreased mRNA expression of the β-subunit of human chorionic gonadotropin (encoded by *CGB*) in placental explants by 35.4%, when compared with controls (Fig. 1i). Analysis of explant culture supernatants matched mRNA data, showing a 25.9% decreased βhCG secretion in the presence of pHPL as well (Fig. 1j). Results from placental explant culture were confirmed in BeWo cells, which were stimulated with forskolin, a compound well described to induce syncytiotrophoblast formation. Both, mRNA expression (Fig. 1k) and secretion of the β-subunit of hCG (Fig. 1l) were strongly increased upon forskolin stimulation, whereas both were significantly impaired in response to pHPL treatment after 48 h. In line with these observations, co-incubation of ADP-stimulated platelets with BeWo cells showed a similar inhibiting effect on forskolin-induced *CGB* expression (Suppl. Fig. 1A).

### Platelet-derived factors do not affect villous trophoblast differentiation

**Fig. 2:**
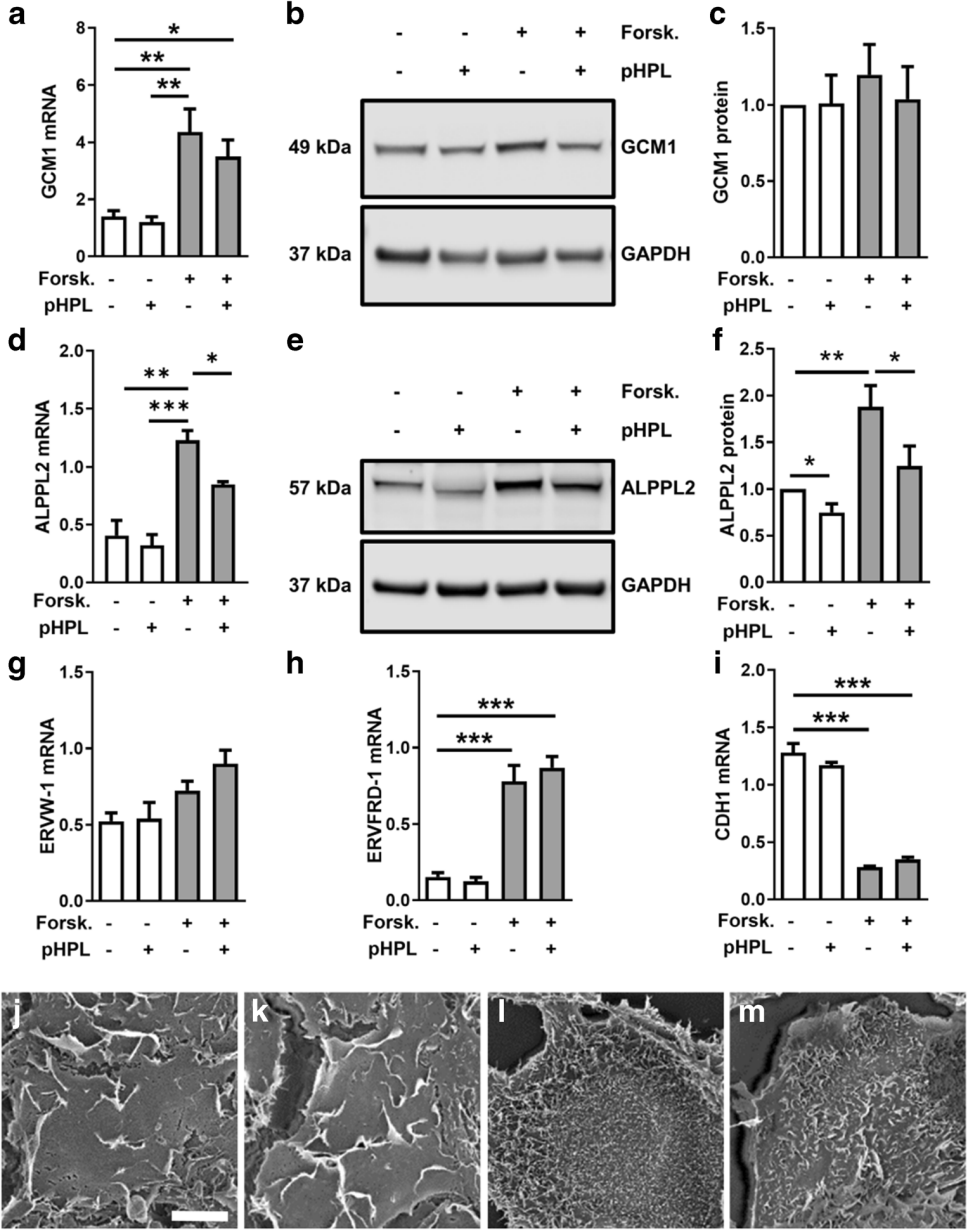
Trophoblast differentiation is not significantly affected by platelet-derived factors. Expression of transcription factor GCM1 mRNA (**a**) and protein levels (**b**, **c**) as well as expression of differentiation marker ALPPL2 mRNA (**d**) and protein levels (**e**, **f**) were analyzed in BeWo cells after forskolin induction (20 μM) in the presence and absence of pHPL after 3 h (*GCM1*) and 48 h (*ALPPL2*). Additionally, expression of markers for trophoblast differentiation and fusion, syncytin-1 (**g**, *ERVW-1*), syncytin-2 (**h**, *ERVFRD-1*), and E-cadherin (**i**, *CDH1*) were analyzed after 48 h. Scanning electron microscopy analyses showed membrane ruffling in vehicle control BeWo cells (DMSO, 0.1% v/v) incubated without (**j**) or with (**k**) pHPL for 48 h. Forskolin-stimulated BeWo cells showed extensive formation of microvilli in absence (**l**) and presence of pHPL (**m**). Data in bar graphs are presented as means ± SEM from six (**a**), four (**c**), or three (all others) independent experiments using different cell passages. Differences between groups were identified using one-way analysis of variance and Tukey’s multiple comparisons test (**a**, **d**, **g–i**). Western blots are representative for four (**b**) and three (**e**) different experiments. For band densitometry (**c**, **f**), controls were set to one and data were tested using one sample *t* test. Scanning electron microscopy images are representative for three different experiments. Scale bar in **j** represents 10 μm. *p ≤ 0.05, **p ≤ 0.01, ***p < 0.001

Since placental βhCG synthesis in vivo occurs in the highly differentiated syncytiotrophoblast, we next tested whether decreased βhCG expression in placental explants and BeWo cells was the consequence of impaired trophoblast differentiation in response to platelet-derived factors. Analyses of the transcription factor glial cells missing homolog (GCM)1, one of the major factors in regulating trophoblast differentiation [8], showed a 3.1-fold increase in mRNA expression after 3 h stimulation with forskolin, which was not significantly impaired in the presence of pHPL (Fig. 2a). On protein level, no significant changes were observed (Fig. 2b, c). Expression of alkaline phosphatase, placental-like 2 (ALPPL2), a marker for biochemical villous trophoblast differentiation, was upregulated after forskolin treatment, and was significantly impaired by pHPL (Fig. 2d). Immunoblot analysis confirmed forskolin-induced upregulation of ALPPL2 on protein level, which was decreased by 33.5% in the presence of pHPL (Fig. 2e and f). The GCM1 downstream targets syncytin-1 (*ERVW-1*, Fig. 2g) and syncytin-2 (*ERVFRD-1*, Fig. 2h, both well-described fusogenic retroviral envelope proteins that trigger trophoblast fusion [9, 10] were upregulated by forskolin after 48 h cultivation, but were not significantly affected by addition of pHPL. Moreover, expression of the cell junction protein E-cadherin (*CDH1*) significantly decreased 4.5-fold as a consequence of forskolin-induced trophoblast fusion, which was, however, not affected by pHPL (Fig. 2i).

Since analysis of these markers did not suggest a substantial effect of pHPL on trophoblast differentiation, we next determined effects on morphological changes in trophoblast differentiation using scanning electron microscopy. Undifferentiated BeWo cells, i.e., incubated with vehicle control alone, frequently showed reef-like membrane ruffles on their surface, irrespective of absence or presence of pHPL (Fig. 2j, k, respectively). In contrast, forskolin stimulation gave rise to formation of closely spaced microvilli (Fig. 2l), which seemed less dense in the presence of pHPL after 48 h (Fig. 2m). In order to substantiate this observation, we analyzed the expression of ezrin, a member of the ezrin-radixin-moesin (ERM) family, which plays a major role in formation and/or maintenance of actin-based cell surface structures [11]. Upon forskolin-induced differentiation, ezrin was significantly upregulated 1.9-fold, whereas the presence of pHPL had no effect on its expression (data not shown). Overall, markers of trophoblast differentiation and fusion, as well as morphological analysis by scanning electron microscopy indicated that impaired hCG synthesis in response to pHPL was not the consequence of impaired syncytialization. This assumption was substantiated by the fact that pHPL addition either at experimental start or after a preceding 48 h forskolin stimulation showed impaired *CGB* expression (Suppl. Fig. 1B).

### Platelet-derived factors do not affect forskolin-induced cAMP/CREB signaling in BeWo cells

**Fig. 3:**
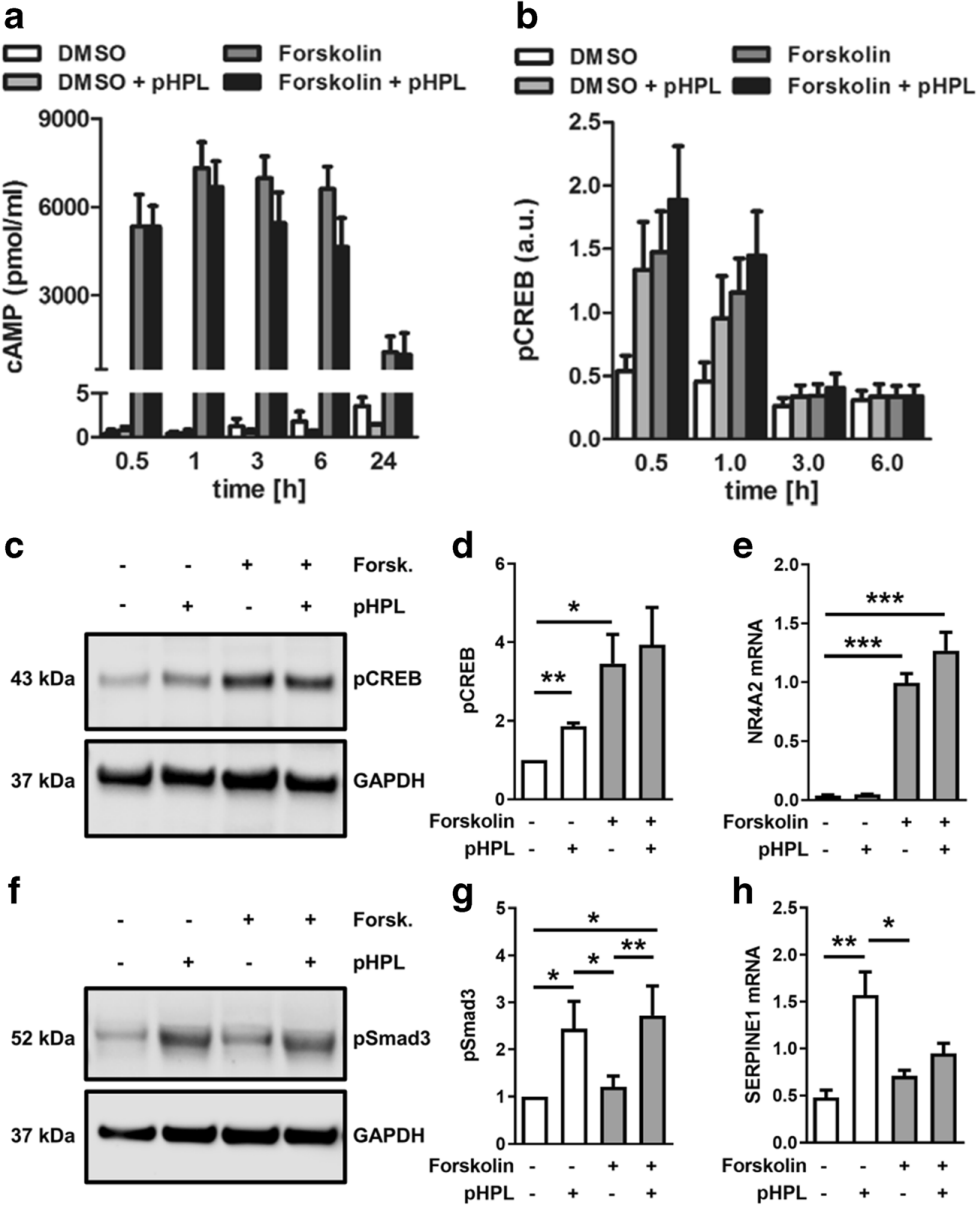
Platelet-derived factors do not affect cAMP/CREB signaling, but activated Smad3 in BeWo differentiation. Levels of intracellular cAMP (**a**) and phosphorylated CREB (**b**) were analyzed by ELISA in forskolin-stimulated (20 μM) and vehicle control (DMSO, 0.1% v/v) BeWo cells in the presence and absence of pHPL at indicated time points. Moreover, phosphorylated CREB (pCREB) was analyzed by immunoblot (**c**) and band densitometry (**d**) after 1 h treatment. Expression of CREB target gene *NR4A2* (**e**) was analyzed in BeWo cells after forskolin induction in the presence and absence of pHPL after 1 h. Phosphorylated Smad3 (pSmad3) was analyzed by immunoblot (**f**) and band densitometry (**g**) after 1 h treatment. Expression of Smad3 target gene *SERPINE1* (**h**) was analyzed in BeWo cells after 48 h. Data in bar graphs **a** and **b** are presented as means ± SEM from five independent experiments using different cell passages and were tested for differences using two-way analysis of variance followed by Tukey’s multiple comparisons test. Western blots are representative for three (**c**) and eight (**f**) different experiments. For band densitometry (**d**, **g**), controls were set to one and data were tested using one sample *t* test. Data in **e** and **h** were analyzed using one-way analysis of variance and Tukey’s multiple comparisons test. *p ≤ 0.05, **p ≤ 0.01, ***p < 0.001

In order to unravel underlying mechanisms, effects of pHPL on forskolin-induced cAMP/CREB signaling were determined in BeWo cells. Forskolin stimulation led to a steep rise in intracellular cAMP after 30 min, which sustained at this level until 6 h (Fig. 3a). Addition of pHPL did not affect the rise in intracellular cAMP neither after 30 min nor between 1 and 6 h of forskolin treatment (Fig. 3a). Since rising cAMP activates the cAMP response element-binding protein (CREB), levels of activated, i.e., phosphorylated-CREB (pCREB) were analyzed in the absence and presence of pHPL. As expected, forskolin stimulation of BeWo cells and subsequent ELISA showed 2.7-fold and 2.5-fold increased phosphorylation of CREB at serine 133 after 30 min and 1 h, respectively, while levels declined to control levels after 3 h (Fig. 3b). Addition of pHPL did not affect the forskolin-induced activation of CREB (Fig. 3b). Interestingly, the presence of pHPL alone, without forskolin stimulation, induced a 2.4-fold and 2.1-fold phosphorylation of CREB after 30 min and 1 h, respectively (Fig. 3b). Data from ELISA were confirmed by immunoblot analysis, showing no significant effect of pHPL on forskolin-induced activation of CREB after 1 h incubation (Fig. 3c, d). Analysis of the expression of Nuclear Receptor Subfamily 4 Group A Member 2 (*NR4A2*), a previously described downstream target of cAMP/CREB signaling [12], showed strong induction in response to forskolin after 1 h, confirming activation of CREB, but did not show a significant difference between presence or absence of pHPL (Fig. 3e). In summary, pHPL treatment did not significantly alter cAMP and pCREB levels in forskolin-induced BeWo differentiation.

### Platelet-derived factors activate Smad3 in BeWo cells

Since platelet-derived factors include a number of factors that regulate growth and differentiation, such as the transforming growth factor (TGF)-β superfamily members TGF-β and bone morphogenetic proteins (BMPs)-2, BMP-4, and BMP-6, we next analyzed effects of pHPL on TGF-β-signaling in forskolin-stimulated BeWo cells. Initial membrane-based immunoblot array screening of the phosphorylation status of eight TGF-β pathway proteins showed that neither forskolin treatment alone nor combined forskolin with pHPL activated Smad1, Smad2, Smad4, and Smad5 (Suppl. Fig. 2 and Suppl. Table 2). Moreover, pHPL did not activate TGF-beta-activated kinase (TAK)1 or the transcription factors ATF2 and c-Jun, whereas c-Fos showed increased activation in presence of pHPL, irrespective of incubation with or without forskolin (Suppl. Fig. 2 and Suppl. Table 2). Additional immunoblot analysis revealed a 2.5-fold increase of phosphorylated Smad3 in the presence of pHPL, while forskolin treatment alone had no effect after 1 h incubation (Fig. 3f, g). Importantly, the activation of Smad3 in response to pHPL stimulation was confirmed by a second antibody clone (Suppl. Fig. 3). Analysis of the well-described Smad3 downstream target plasminogen activator inhibitor (PAI)-1 [13], encoded by the gene *SERPINE1*, showed a 3.3-fold upregulation after treatment with pHPL alone, while combined administration of forskolin and pHPL impaired this effect after 48 h (Fig. 3h). These data suggest that platelet-derived factors activate TGF-β-signaling through Smad3 activation in BeWo cells.

### Inhibitors of TGF-β/Smad3-signaling do not restore impaired βhCG synthesis

**Fig. 4:**
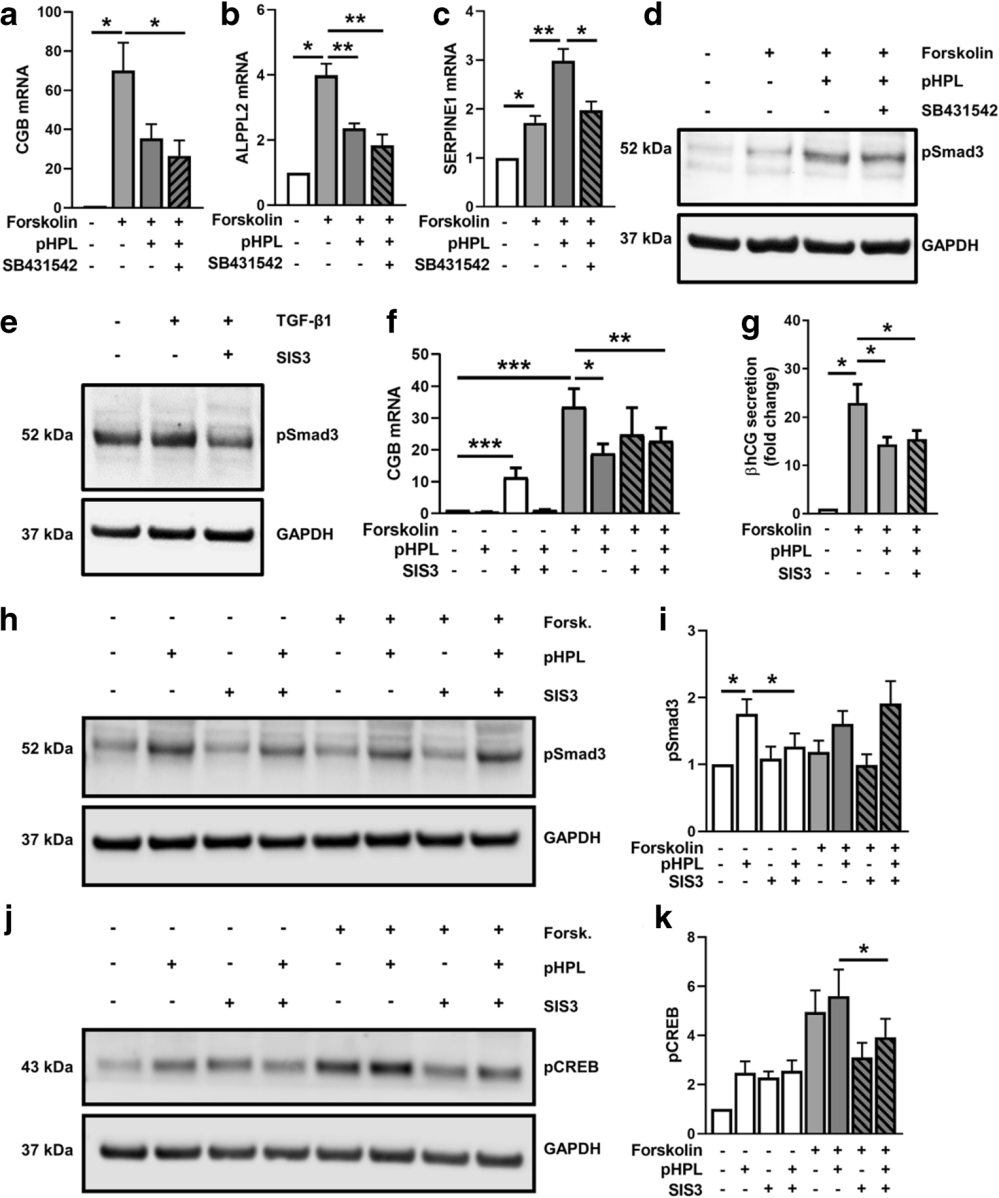
Inhibitors of TGF-β/Smad3-signaling do not restore impaired βhCG synthesis. BeWo cells were pre-incubated with SB431542 at final concentrations of 10 μM for 2 h. Thereafter, *CGB* (**a**), *ALPPL2* (**b**), and *SERPINE1* (**c**) expressions were analyzed in BeWo cells after forskolin induction (20 μM) in the presence and absence of pHPL and with or without SB431542 (10 μM) after 48 h. Efficiency of SB431542 (**d**) and SIS3 (**e**, 10 μM) was evaluated by immunoblots for phosphorylated Smad3 (pSmad3) in BeWo cells, after pre-incubation with the inhibitors for 1 h and subsequent stimulation with or without pHPL or TGF-β1 (10 nM) for 1 h. Expression of *CGB* (**f**) and secretion of βhCG (**g**) were analyzed in BeWo cells after forskolin induction (20 μM) in presence and absence of pHPL and with or without SIS3 (10 μM) after 48 h. Immunoblots and band densitometry for pSmad3 (**h**, **i**) and pCREB (**j**, **k**) were analyzed in BeWo cells after 1 h pre-incubation with SIS3 (10 μM) and subsequent forskolin stimulation in presence and absence of pHPL for 1 h. Data in bar graphs are presented as means ± SEM from five (**f**) and three (all others) independent experiments using different cell passages. Data in **a**–**c** and **f** and were tested for differences using one-way analysis of variance followed by Tukey’s multiple comparisons test. Western blots are representative for three (**d**, **e**) and five (**h, j**) different experiments. For data analysis of secreted βhCG (**g**) and band densitometry (**i**, **k**), controls were set to one and data were tested using one sample *t* test. *p ≤ 0.05, **p ≤ 0.01, ***p < 0.001

To determine whether impaired βhCG synthesis in response to platelet-derived factors was a result of TGF-β-signaling and Smad3 activation, we next used SB431542, a selective inhibitor of the TGF-β type I receptor (TGFBR1). While pHPL again decreased *CGB* (Fig. 4a) and *ALPPL2* (Fig. 4b) expressions in forskolin-stimulated BeWo cells by 50% and 40%, respectively, an addition of SB431542 did not reverse this effect. In contrast, pHPL-induced *SERPINE1* upregulation was significantly blocked by SB431542, suggesting sufficient efficiency of the inhibitor (Fig. 4c). However, administration of SB431542 did only partially inhibit pHPL-induced activation of Smad3 (Fig. 4d), arguing against the involvement of TGFBR1 in impaired *CGB* expression.

Next, we used SIS3, a specific inhibitor of TGF-β/Smad3 signaling [14], which showed appropriate efficiency to decrease TGF-β-induced Smad3 phosphorylation in BeWo cells (Fig. 4e). However, SIS3 did not block the pHPL-mediated decrease of βhCG mRNA expression (Fig. 4f) and secretion (Fig. 4g) in forskolin-stimulated BeWo cells. Surprisingly, SIS3 administration alone, i.e., without addition of forskolin and/or pHPL, showed a substantial 11-fold induction of *CGB* expression, when compared with the non-stimulated control (Fig. 4f). This led us to further investigate the effects of SIS3 on both Smad3 and CREB activation in forskolin-stimulated BeWo cells, which were incubated with or without pHPL. Accordingly, phosphorylation of Smad3 increased in the presence of pHPL in non-stimulated cells and was blocked almost to control levels by SIS3 after 1 h (Fig. 4h, i). However, in forskolin-stimulated cells, SIS3 did not inhibit pHPL-induced Smad3 phosphorylation (Fig. 4h, i). Although described as specific Smad3 inhibitor, SIS3 showed considerable effects on CREB phosphorylation. In non-stimulated cells, SIS3 per se induced CREB phosphorylation, while in forskolin-stimulated cells SIS3 administration considerably inhibited phosphorylation of CREB (Fig. 4j, k). Together, these data suggest an interference between Smad3 and CREB signaling in BeWo cells.

### CREB-binding proteins are required for efficient βhCG synthesis

**Fig. 5:**
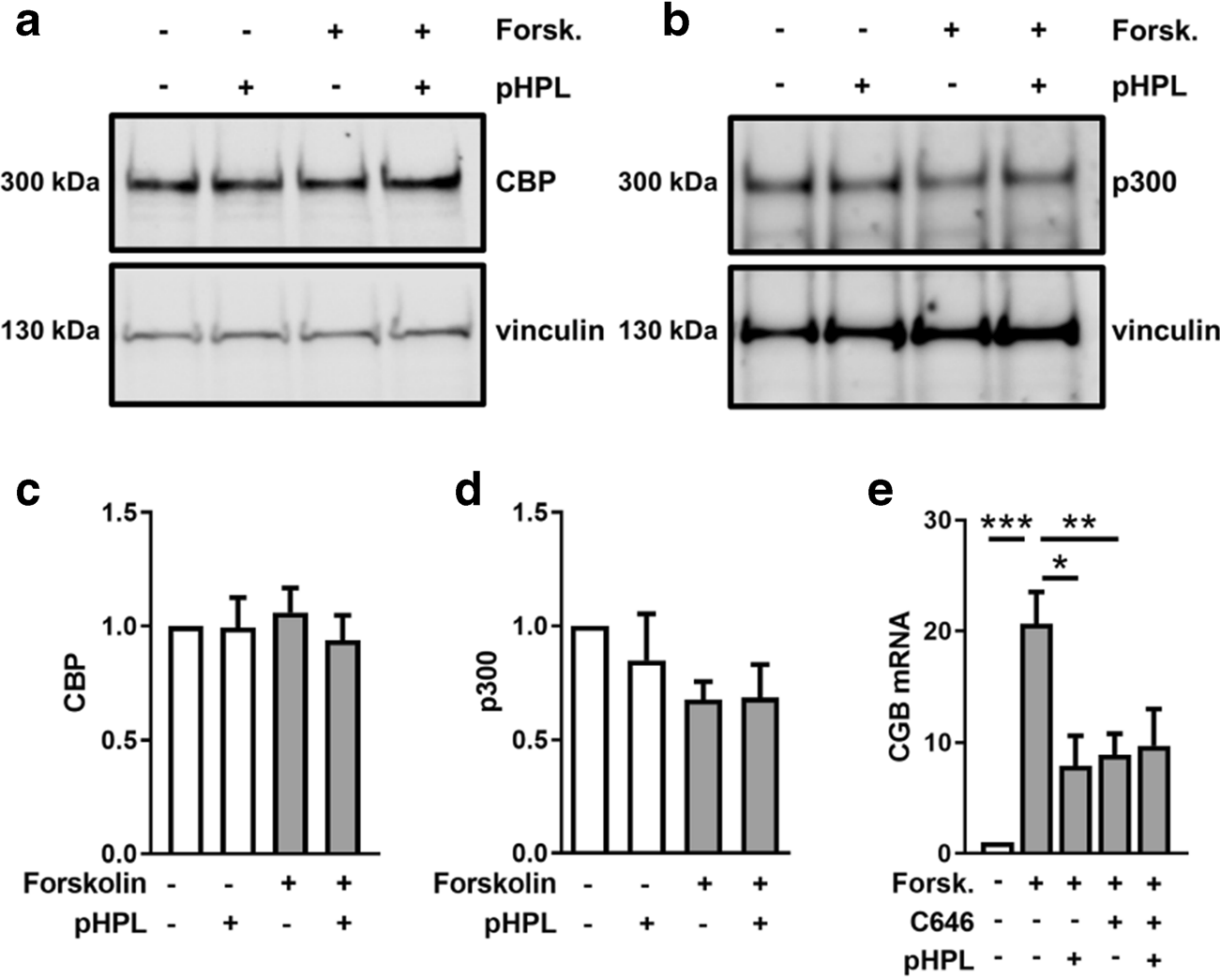
Inhibition of CBP/p300 impairs *CGB* expression in BeWo cells. Immunoblots and band densitometry for CBP (**a**, **c**) and p300 (**b**, **d**) were analyzed in BeWo cells after forskolin stimulation (20 μM) in the presence and absence of pHPL after 1 h. *CGB* expression (**e**) was analyzed in BeWo cells after forskolin induction (20 μM) in presence and absence of C646 (20 μM) after 24 h. Data in **c** and **d** are presented as means ± SEM from four, and those in **e** from three independent experiments using different cell passages. Western blots are representative for four different experiments. For data analysis of band densitometry (**c**, **d**), controls were set to one and data were tested using one sample *t* test. Data in **e** were tested for differences using one-way analysis of variance followed by Tukey’s multiple comparisons test. ***p < 0.001

The transcription co-activators CREB-binding protein (CBP) and p300 (also referred to as EP300) have been implicated to play essential roles in both, Smad- and CREB-driven gene expression [15]. Thus, we analyzed CBP and p300 protein levels, to determine whether impaired βhCG synthesis was a result of deregulated co-activators. However, neither CBP (Fig. 5a, c) nor p300 (Fig. 5b, d) protein levels were significantly changed in the presence of pHPL. Forskolin stimulation showed a trend to decrease p300 levels by 30%, which however did not reach statistical significance. Next, we tested whether CREB-binding proteins are required for *CGB* expression. Administration of C646, a selective inhibitor of CBP and p300 [16], significantly impaired forskolin-induced *CGB* expression in BeWo cells by 57.1% after 24 h (Fig. 5e). Finally, we tested whether or not there is an additive effect, when cells were co-treated with the CBP/p300 inhibitor and pHPL. While incubation with C646 or pHPL alone showed similar inhibiting effects, co-incubation of cells with C646 and pHPL did not show an effect beyond the p300/CPB inhibition with C646 alone (Fig. 5e).

## Discussion

Here, we provide evidence that platelet-derived factors impair placental βhCG synthesis. Although trophoblast plugs in uterine arteries obstruct maternal arterial blood flow to the developing placental chorionic tissue during the first trimester of human pregnancy, maternal platelets may pass through narrow intertrophoblastic gaps that have been suggested to enable initial microvascular flux by 7 weeks of gestation [1]. We show platelets on the surface of placental villi from 5 weeks gestation onwards, where they either adhered directly on the apical surface of the syncytiotrophoblast or on initial perivillous fibrinoid. Moreover, we detected platelets between the villous cytotrophoblast and the syncytiotrophoblast layer. These observations suggest that platelets gain access to the intervillous space early in first trimester, and moreover, that they are involved in re-epithelialization of damaged syncytiotrophoblast areas and contribute to perivillous fibrin deposition. On the basis of these assumptions, it is tempting to speculate whether or not alterations in the intensity of maternal plasma flow may affect the degree of adherence and activation of maternal platelets at the maternal-fetal interface. Once uteroplacental blood flow is completely established, turbulences may cause shear stress and subsequent injury to the trophoblast layer. Damaged regions of villi may become denuded of syncytiotrophoblast, and exposure of extracellular matrix molecules may induce the maternal coagulation cascade, eventually leading to depositions of fibrin-type fibrinoid at these sites of injury [17]. Consequently, the syncytial epithelium may become re-established over the fibrin matrix by cytotrophoblasts which proliferate, differentiate, and fuse [18, 19].

Our observation of maternal platelets in intercellular spaces of HLA-G-positive trophoblasts in anchoring parts of cell columns may represent a yet unidentified way how platelets can enter the early intervillous space. Moreover, we detected maternal platelets in fragmentary trophoblast plugs of uterine blood vessels, which is in good agreement with a previous study by Sato et al. [2]. From our staining, it is not possible to assess whether or not they get activated by this passage. However, previous co-culture of isolated extravillous trophoblasts with platelets led to externalization of P-selectin to the surface of adherent platelets, suggesting they had been activated [2]. Hence, adherence and activation of maternal platelets in narrow intercellular gaps of trophoblast plugs or cell columns may be followed by degranulation and release of granule-stored factors, which then could easily be transported into the intervillous space by the maternal ultrafiltrate, blood plasma [20].

Villous trophoblast differentiation and fusion are regulated by a wide panel of growth factors and cytokines [21], some of which, like epidermal growth factor (EGF) and TGF-β, are abundantly found in platelet granules and pHPL [22]. However, our data suggest that morphological differentiation, if at all, is only marginally impaired in the presence of platelet-derived factors. The observation of impaired hCG synthesis despite unchanged differentiation argues for different regulatory mechanisms, which may be interconnected, but may not necessarily be regulated through the same pathways. This has previously been suggested by Johnstone et al., who showed that EGF treatment of primary trophoblasts inhibited hCG secretion, but at the same time stimulated syncytialization [23]. Moreover, hCG synthesis has been reported in forskolin-stimulated BeWo cells that were hindered to fuse by treatment with the protein kinase inhibitor H-89 [24], again arguing for different regulatory mechanisms.

In the present study, we demonstrate that hallmarks of trophoblast fusion, such as reduction in E-cadherin expression [25], as well as upregulation of syncytins [9] and microvilli formation were not significantly altered by platelet-derived factors. However, it should be stressed that sample size of cell culture experiments in this study is too small to identify minor but statistically significant differences between treatments. Since neither morphology nor differentiation markers significantly changed, we suggest that impaired βhCG synthesis in response to platelet-derived factors was not the consequence of impaired syncytialization. Thus, platelet-derived factors may directly act on the syncytiotrophoblast—an assumption which is supported by the fact that βhCG synthesis was impaired by platelet-derived factors regardless of adding them at experimental start or after 48 h forskolin stimulation, when syncytialization already had occurred (Suppl. Fig. 1B). This is in good agreement with a study by Song et al., showing a reduction in hCG production, when adding TGF-β to primary trophoblasts after 4-day culture, after transformation of cyto- to syncytiotrophoblast has taken place [26].

Indeed, TGF-β has previously been described to decrease a number of fundamental trophoblast-derived pregnancy hormones, including progesterone and estradiol as well as human placental lactogen (hPL) and hCG [26–28]. Moreover, TGF-β-Smad signaling has been shown to decrease expression of GCM1 and ERVW-1 in villous explants and BeWo cells [29]. Though platelets are a major source of TGF-β, our inhibitor experiments using SB431542 rather argue against the involvement of TGF-β type I receptor in this process. However, it is important to stress that forskolin abrogates the inhibiting action of SIS3 on Smad3 activation, and, moreover, SIS3 per se activates CREB. If this observation is just the result of unspecific side effects by SIS3 or some upstream interaction between Smad3 and CREB signaling remains unclear at this point. Importantly, increased levels of cAMP, which is a key messenger of many hormones and neuropeptides, have been shown to antagonize the effects of TGF-β [15].

**Fig. 6:**
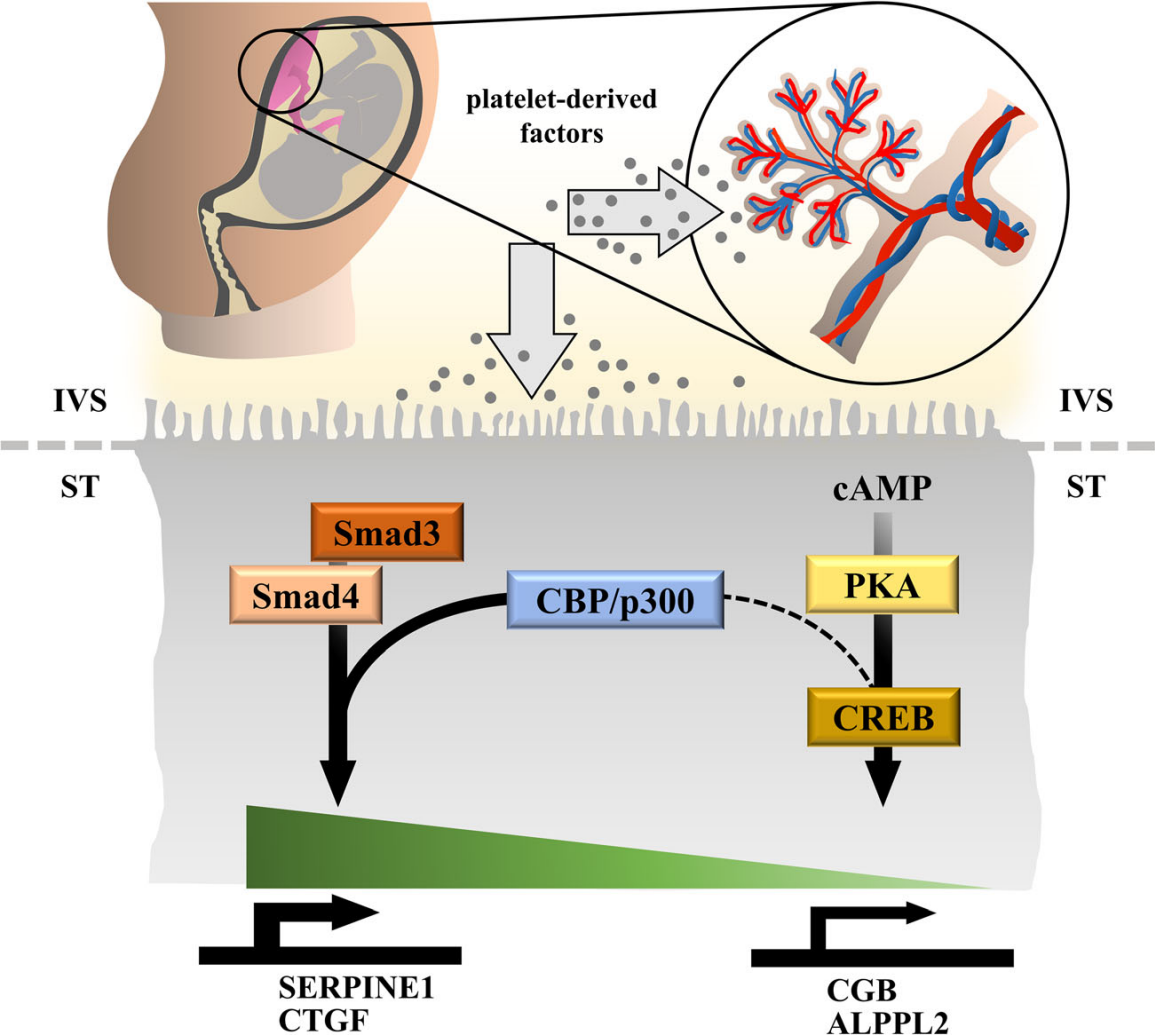
Proposed concept how platelet-derived factors impair placental βhCG synthesis. Activation of maternal platelets at the maternal-fetal interface is followed by degranulation of granule-stored factors, which then could easily be transported into the intervillous space (IVS) where they can act on the syncytiotrophoblast (ST). Activation of Smad signaling in response to platelet-derived factors induces expression of Smad3 targets, such as plasminogen activator inhibitor 1 (PAI-1, encoded by *SERPINE1*) and connective tissue growth factor (*CTGF*). At the same time, activation of Smad-signaling abrogates CREB-dependent expression of βhCG (*CGB*) and alkaline phosphatase, placental-like 2 (*ALPPL2*) by sequestrating the transcriptional co-activators CBP/p300

Previous experiments with human dermal fibroblasts revealed a functional interaction between cAMP/CREB and TGF-β signaling, resulting in a strong suppressive effect of both forskolin and the membrane-permeable cAMP analog dibutyryl-cAMP on extracellular matrix (ECM)-related genes, including collagen type I, connective tissue growth factor (CTGF), TIMP metallopeptidase inhibitor 1, and PAI-1. This suppressive effect has been explained by sequestration of the co-activators CBP and p300 by activated CREB, as shown by elegant experiments using a mammalian two-hybrid system [15]. Our observation of impaired PAI-1 mRNA expression in response to combined administration of forskolin and pHPL (Fig. 3h) may thus be explained by interference of cAMP/CREB and TGF-β signaling. Since the amount of nuclear CBP/p300 is limited, formation of CREB-CBP/p300 complexes may reduce the amount of CBP/p300 available to Smad3 and vice versa. Thus, it is tempting to speculate that platelet-derived TGF-β activates Smad3, which sequestrates co-activators CBP and/or p300 and in turn reduces their availability to CREB, leading to reduced βhCG synthesis in forskolin-stimulated trophoblasts (Fig. 6). The interaction of Smad3 and CBP and/or p300 has previously been shown by co-immunoprecipitation of overexpressed FLAG- or Myc-tagged Smad3 and co-activators, respectively [30]. Unfortunately, co-immunoprecipitation of Smad3 and CBP/p300 failed in our cells, which may be explained by the fact that endogenous levels of involved proteins are too low for successful pull-down.

However, our experiments with the selective CBP/p300 inhibitor C646 showed significantly impaired forskolin-induced *CGB* expression, which was not further impaired after simultaneous C646 and pHPL treatment, suggesting that the amount of CBP/p300 available to CREB is indeed a rate-limiting factor for trophoblastic βhCG synthesis. Of note, previous immunostainings showed activated CREB and CBP strongly expressed in nuclei of the syncytiotrophoblast, whereas p300 seems to be primarily expressed in cytotrophoblasts but could also be detected in a low number of syncytial nuclei [31]. Smad3 is considerably expressed in isolated primary extravillous trophoblasts, whereas it has been detected only marginally in the villous trophoblast population, suggesting that TGF-β-mediated Smad-signaling is largely absent in the floating placental epithelium under homeostatic conditions [32]. However, TGF-β Smad-dependent signaling through activation of TGF-β-receptors has been previously shown by others in first trimester placental explant culture [29, 33] and our own current data demonstrate that platelet-derived factors substantially induced PAI-1, a well-described downstream target of TGF-β/Smad-signaling [13]. Upregulation of placental PAI-1, as a consequence of perivillous platelet aggregation, may promote augmented deposition of fibrin-type fibrinoid by inhibiting both tissue plasminogen activator (tPA) and urokinase-type plasminogen activator (uPA). Importantly, deposition as well as continuous clearance of fibrin-type fibrinoid are normal events in human placenta throughout pregnancy [34]. A disbalanced turnover of placental fibrin-type fibrinoid has been suggested for pregnancies complicated by pregnancy-induced hypertension [35, 36] and maternal diabetes mellitus [37].

Impaired placental hCG secretion in response to platelet-derived factors may have serious consequences on pregnancy outcome, since multiple important steps of early placentation, including trophoblast proliferation, differentiation, and invasion, are regulated by this hormone [38]. In line with this assumption, previous studies suggest that low hCG concentrations in late first trimester may be associated with increased risk to develop preeclampsia later on in pregnancy [39, 40]. Interestingly, low hCG concentrations very early in pregnancy have been associated with a subsequent risk of preeclampsia as well. This has been shown in a prospective follow-up study of pregnancies conceived after IVF. Accordingly, maternal concentrations of hCG on day 12 after embryo transfer were inversely associated with the risk for severe preeclampsia in a dose-dependent manner [41]. Whether antiplatelet therapy in early gestation abolishes impaired hCG production by blocking platelet activation at the maternal-fetal interface remains speculative. Amongst anticoagulants, low-dose aspirin administration in early pregnancy is currently controversially discussed to have beneficial effects on pregnancy outcome. Meta-analyses of randomized controlled trials suggest that aspirin reduces the risk of preterm preeclampsia, but not term preeclampsia, and only when it is initiated at ≤ 16 weeks of gestation and at a daily dose of ≥ 100 mg [42]. However, compliance with treatment and individual response may also contribute to the effectiveness of aspirin therapy [43].

In summary, our study suggests that maternal platelets can pass through intercellular clefts of extravillous trophoblast plugs and cell columns, enabling entrance into the early intervillous space. By the time of platelet activation and degranulation, platelet-derived factors impair placental βhCG production, without substantially affecting morphological and biochemical differentiation of villous trophoblasts.

## Materials and methods

### Human placenta tissue samples

The study was approved by the ethical committee of the Medical University of Graz (26-132 ex 13/14 and 31-019 ex 18-19). First trimester placental tissues were obtained between weeks 5 and 12 of gestation with written informed consent from women undergoing legal elective pregnancy terminations.

### Immunohistochemistry and immunohistochemical double staining

Human formalin-fixed paraffin-embedded (FFPE) first trimester placenta tissues (*n* = 31, mean gestational week 8.01 ± 2.08, see Suppl. Table 1) were cut (5 μm) and mounted on Superfrost Plus slides. After deparaffinization, slides were subjected to antigen retrieval by boiling sections in Epitope Retrieval Solution pH 9.0 (Novocostra, Leica) for 7 min at 120 °C. Thereafter, sections were stained using a staining robot (Autostainer 360; Thermo Fisher Scientific) with primary antibodies as indicated in Suppl. Table 3 using the UltraVision Large Volume Detection System HRP Polymer Kit (Thermo Fisher Scientific) as previously described [3, 44].

Immunohistochemical double staining was performed with the Multivision Polymer Detection system using mouse monoclonal anti-HLA-G and rabbit polyclonal anti-vWF antibodies using dilutions as indicated in Suppl. Table 3 according to the protocol previously described [45].

### Placental explant culture

Placental villous tissue from human first trimester (*n* = 14, mean gestational week 8.26 ± 0.45) was thoroughly rinsed in buffered saline and dissected into small pieces of approximately 5 mg moist mass as described previously [46]. Placental explants were cultured in 6 well dishes (Nunc) and 4 ml/well DMEM (including low glucose, pyruvate, l-glutamine) supplemented with 10% FCS (HyCloneTM; Gibco) and penicillin/streptomycin (Gibco), in a hypoxic workstation (BioSpherix; Redfield, NY, USA) under 2.5% oxygen for indicated durations at 37 °C. For treatments, culture medium was supplemented with 10% pooled human platelet lysate (pHPL) and heparin (Merck, Darmstadt, Germany). Heparin served as an anticoagulant and was added to pHPL-supplemented media as well as to the controls at a final concentration of 2 U/ml. pHPL was produced at the Department of Transfusion Medicine, Paracelsus Medical University of Salzburg as previously described [47]. In brief, each batch of pHPL was prepared by pooling ten expired buffy-coat-derived platelet concentrates (40 blood donations) after lysis by several freeze-thaw cycles at − 30 /37 °C and a final centrifugation step (4000 × *g* 15 min) to deplete platelet fragments.

### Culture of BeWo cell line

BeWo cells were purchased from the European Collection of Cell Cultures (ECACC) and were cultured as previously described [48]. In brief, BeWo cells were cultured in DMEM/F12 (1 : 1, Gibco, life technologies; Paisley, UK) supplemented with 10% FCS (Gibco), penicillin/streptomycin (Gibco), and l-glutamine (Gibco) in a humidified atmosphere of 5% CO_2_ at 37 °C. Cells between passage 10 and 20 were used for in vitro experiments. In case of forskolin treatment, cells (2 × 10^5^ cells/well) were plated in 12-well dishes (Nunc Lab-Tek, Thermo Fisher; NY, USA) one day prior to experimental start in the above described culture medium. Next day, the culture medium was exchanged with a medium including forskolin (Tocris, Bio-techne, Abingdon, UK) at a final concentration of 20 μM (20 mM stock in DMSO) supplemented with or without 10% pHPL. DMSO served as the vehicle control for forskolin and was applied at a final concentration of 0.1% (v/v).

For inhibitor experiments, BeWo cells were pre-incubated for 1 h at the following concentrations: 10 μM for SB431542 (Sigma-Aldrich), 10 μM for SIS3 (Tocris, Bio-techne, UK), and 20 μM for C646 (Sigma). After pre-incubation, culture medium was exchanged with medium supplemented with or without 20 μM forskolin or solvent control, 10% pHPL and inhibitors, respectively, as indicated. Cell culture experiments were repeated three times or more (as indicated in the figure legends), using different cell passages.

### qPCR analysis

Total RNA was isolated with peqGOLD TriFast (VWR, Radnor, Pennsylvania, USA) according to the manufacturer’s instructions. Quality check was followed by reverse transcription of 1 μg total RNA per reaction using High-Capacity cDNA Reverse Transcription Kit (Applied Biosystems) according to manufacturer’s manual. qPCR was performed with Universal SYBR Green Supermix (Bio-Rad, Hercules, CA, USA) using a Bio-Rad CFX96 cycler as previously described [49] with specific primers and the run protocol shown in Suppl. Table 4. Cq values were automatically determined using single thresholds and normalized expression (ΔΔCq analysis) was automatically generated by the CFX Manager 2.0 Software (Bio-Rad). The expression of *GAPDH*, *CYC1*, and *YWHAZ* was used as reference, according to a previous evaluation of housekeeping genes in placental tissue [50].

### Measurement of secreted βhCG

Culture media were collected at indicated time points and centrifuged at 1500 rpm for 5 min. Supernatants were stored at − 80 °C and subjected in groups to routine immunoassay analyses (Dimension Xpand; Dade Behring Inc., Deerfield, Illinois). Obtained values were normalized to total cell and tissue protein, respectively, and controls were set to 1.

### Immunoblotting

After incubations, cells and placental explants were washed with PBS and homogenized in RIPA buffer (Sigma-Aldrich) including protease inhibitor cocktail (Roche Diagnostics; Mannheim, Germany) and phosSTOP (Roche Diagnostics). Homogenates were centrifuged at 8000 × *g* and 4 °C for 10 min. Concentration of total protein was determined in clear supernatants according to Lowry method. Twenty micrograms total protein were applied to precast 10% Bis-Tris or 3–8% Tris-Acetate gels (NuPAGE, Novex; lifetechnologies). Proteins were blotted on a 0.45-μm nitrocellulose membrane (Hybond, Amersham Biosciences, GE Healthcare Life Sciences, Little Chalfont, UK) and blotting efficiency was determined with Ponceau staining (Ponceau S solution, Sigma Aldrich). Membranes were cut in horizontal strips at molecular weight ranges for target proteins. Primary antibodies were diluted as described in Suppl. Table 3 and incubated on membranes overnight at 4 °C. HRP conjugated goat anti-mouse and goat anti-rabbit IgG (1 : 3000, Bio-Rad) were used as secondary antibodies and incubated on membranes for 2 h at RT. Immunodetection was performed with a chemiluminescent immunodetection kit (Amersham ECL Prime Western blotting detection Reagent) according to the manufacturer’s instructions. Images were acquired with FluorChem Q System (Alpha Innotech, Cell Bioscienes, Santa Clara, CA, USA) and iBright CL 1000 Imaging System (Thermo Fischer Scientific) and band densities were analyzed with Li-Cor Image Studio Lite 5.2. Results are presented as a ratio of band densities of target protein and reference proteins GAPDH and vinculin with control samples set to one.

### Scanning electron microscopy

BeWo cells were seeded in 12-well culture dishes containing round cover slips (15 mm, Thermo Fischer Scientific) at a density of 2 × 10^5^ cells/well. Next day, cells were stimulated with forskolin (20 μM) and incubated in the absence or presence of pHPL as described above. DMSO served as the vehicle control for forskolin and was applied at a final concentration of 0.1% (v/v). After 48 h culture, cells were washed in PBS and fixed in 2% paraformaldehyde and 2.5% glutaraldehyde solved in 0.1 M sodium phosphate buffer (pH 7.4). After rinsing in sodium phosphate buffer, specimens were post-fixed in 2% osmium tetroxide (Electron Microscopy Sciences) solved in 0.1 M sodium-phosphate buffer (pH 7.4) and rinsed again. Each step was performed for 30 min at RT. After dehydration in a graded series of ethanol and critical point drying (CPD 030; Bal-Tec, Balzers, Liechtenstein), samples were sputter coated with gold palladium (SCD 500; Bal-Tec, Balzers, Liechtenstein). Images were acquired using a Zeiss Sigma 500 field emission scanning electron microscope (Zeiss, Oberkochen, Germany), operated at an acceleration of 3 kV with an Everhart-Thornley-secondary electron detector.

### Analysis of intracellular cAMP and pCREB levels

cAMP levels were measured in BeWo cell lysates in duplicates without acetylation step using the Direct cAMP ELISA kit (Enzo Life Sciences, Switzerland), according to the manufacturer’s manual. pCREB levels were measured in BeWo cell lysates using Human Phospho-CREB (S133) DuoSet IC ELISA (R&D Systems, Bio-techne, Abingdon, UK) according to the manufacturer’s protocol.

### Statistical analysis

Data were analyzed using GraphPad Prism Version 8.1.0 and are presented as means ± SEM. Data were subjected to Normality test (D’Agostino and Pearson omnibus normality test) and equal variance test. In case of normally distributed data differences between groups were tested using two-tailed *t* test. Otherwise Wilcoxon’s signed rank test was applied. For multiple comparison procedure, one-way analysis of variance was followed by Tukey’s multiple comparisons test to isolate groups that differ from the others. One sample *t* test was used when controls were set as 1. A *p* value of less than 0.05 was considered statistically significant.

## Electronic supplementary material

ESM 1 (PNG 23 kb)

High Resolution (TIF 44 kb)

ESM 2 (PNG 64 kb)

High Resolution (TIF 209 kb)

ESM 3 (PNG 91 kb)

High Resolution (TIF 391 kb)

ESM 4 (DOCX 33 kb)
