# Supplementary figures and images for: Platelet-derived factors impair placental chorionic gonadotropin beta-subunit synthesis
Source: J Mol Med (Berl). 2019 Dec 20;98(2):193–207. doi: 10.1007/s00109-019-01866-x (PMC7007904; doi:10.1007/s00109-019-01866-x)

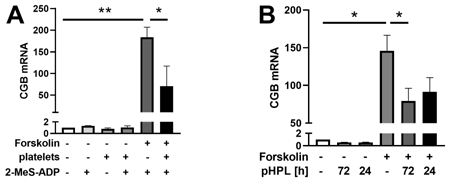

Supplement: Supplementary file 1 — (PNG 23 kb) [file 109_2019_1866_Fig1_ESM.png]

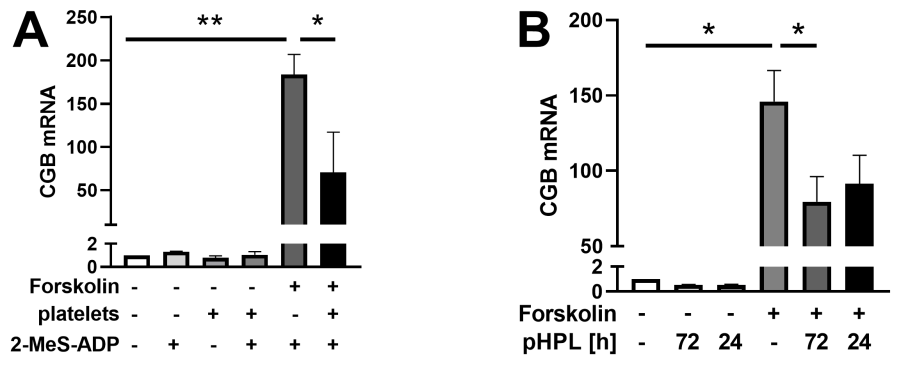

Supplement: Supplementary file 2 — High Resolution (TIF 44 kb) [file 109_2019_1866_MOESM1_ESM.tif]

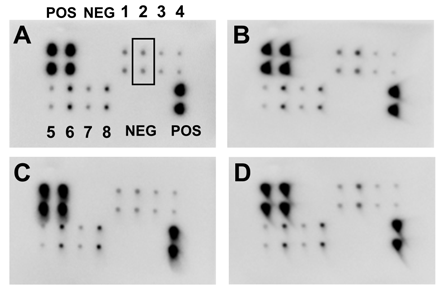

Supplement: Supplementary file 3 — (PNG 64 kb) [file 109_2019_1866_Fig2_ESM.png]

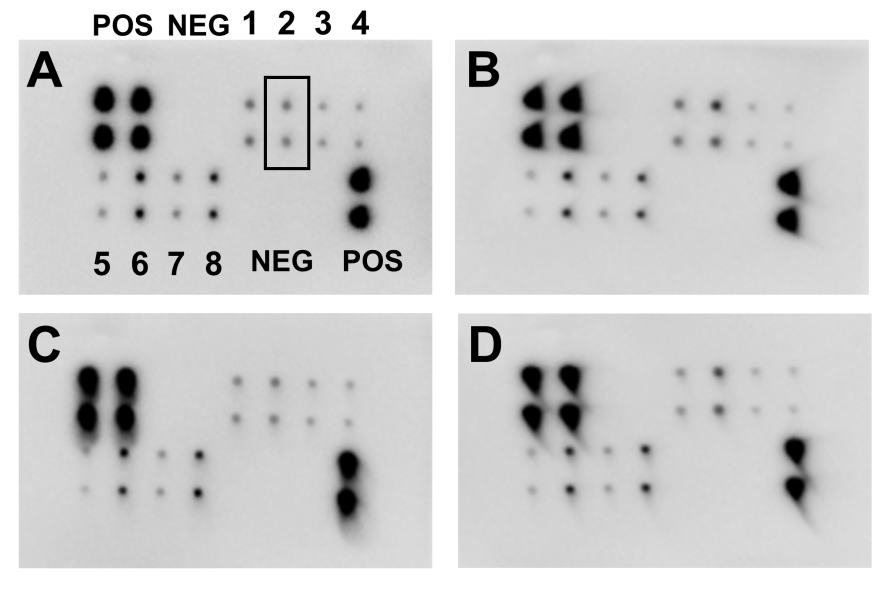

Supplement: Supplementary file 4 — High Resolution (TIF 209 kb) [file 109_2019_1866_MOESM2_ESM.tif]

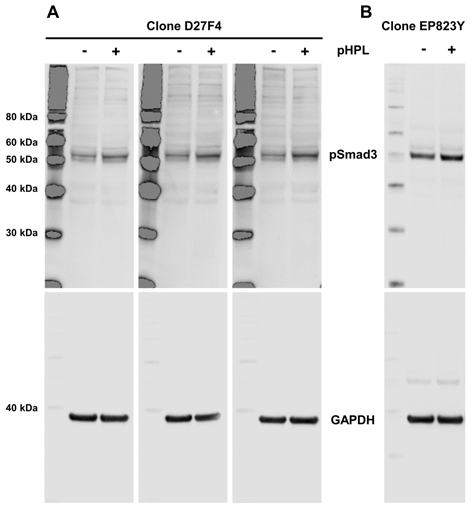

Supplement: Supplementary file 5 — (PNG 91 kb) [file 109_2019_1866_Fig3_ESM.png]

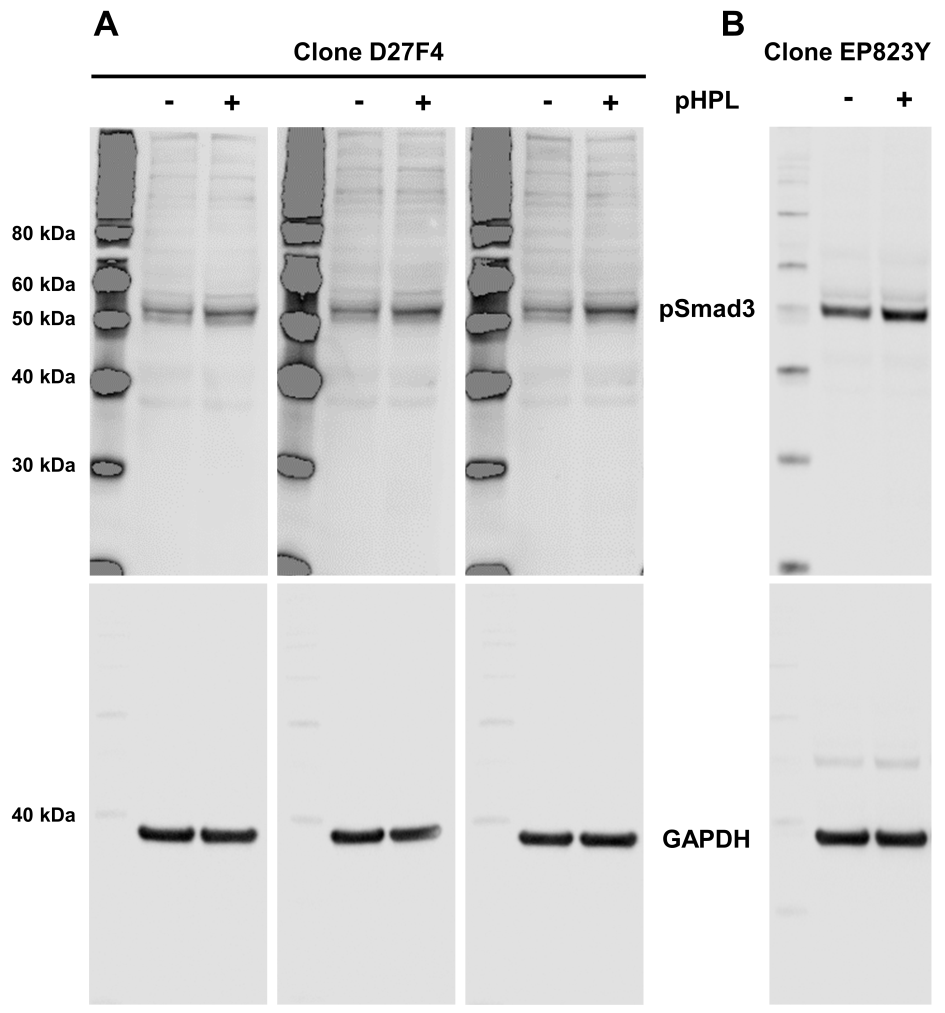

Supplement: Supplementary file 6 — High Resolution (TIF 391 kb) [file 109_2019_1866_MOESM3_ESM.tif]
