# Supplementary material for: Platelet-derived factors impair placental chorionic gonadotropin beta-subunit synthesis
Source: J Mol Med (Berl). 2019 Dec 20;98(2):193–207. doi: 10.1007/s00109-019-01866-x (PMC7007904; doi:10.1007/s00109-019-01866-x)
Supplement: Supplementary file 7 — (DOCX 33 kb) [file 109_2019_1866_MOESM4_ESM.docx]

**Supplements**

**Figure legends to supplemental figures**

**Supplemental figure 1**

*Activated platelets impair CGB expression in BeWo, and impaired CGB expression is independent of the differentiation status*

(**A**) *CGB* expression was analysed in forskolin- (20µM) and vehicle control (DMSO) stimulated BeWo cells, which were incubated with or without human platelets for 48h. Where indicated, platelets were activated with the stable ADP analogue 2-Methylthioadenosine 5'-diphosphate (2-MeS-ADP, 100nM). (**B**) *CGB* expression was analysed in forskolin- and vehicle control (DMSO) stimulated BeWo cells either after pHPL addition at experimental start (72h total incubation) or pHPL addition after 48h pre-incubation with forskolin (24h incubation with pHPL) or DMSO alone. Data are presented as means ± SEM from three independent experiments using different cell passages and three different platelet donors. Data were tested for differences using One-way analysis of variance followed by Tukey's multiple comparisons test. *p≤0.05, **p≤0.01

**Supplemental figure 2**

*Human TGF beta Pathway Phosphorylation Array*

BeWo cells were incubated with solvent control DMSO alone (**A**), with DMSO and pHPL (**B**), with forskolin alone (**C**), or with forskolin and pHPL (**D**) for 1h. Thereafter, cell lysates were subjected to membrane-based immunoblot array screening of the phosphorylation-status of eight TGF-β pathway proteins, including ATF2 (**1**), C-Fos (**2**, highlighted by the rectangle), c-Jun (**3**), Smad1 (**4**), Smad2 (**5**), Smad4 (**6**), Smad5 (**7**) and TAK1 (**8**). Positive control and negative control spots are indicated by POS and NEG, respectively. Increased levels of phosphorylated C-Fos were detected in cells incubated in the presence of pHPL.

**Supplemental figure 3**

*Detection of pHPL-induced Smad3 activation by two different antibody clones*

Phosphorylated Smad3 (pSmad3) was analyzed in BeWo cells treated with or without pHPL for 1h. pSmad3 was detected either with monoclonal rabbit anti-pSmad2(S465/467)/pSmad3(S423/425) antibody (Clone D27F4) (**A**), or with monoclonal rabbit anti-pSmad3(S423/425) antibody (Clone EP823Y) (**B**). Three upper panels in **A** show Western blots performed with anti-pSmad2(S465/467)/pSmad3(S423/425) antibodies (Clone D27F4) from three different purchase dates. Both antibody clones detected a single band, which increased in intensity when cells were treated with pHPL.

**Supplemental Materials and Methods**

*Isolation of human platelets*

Citrated whole blood samples from healthy donors were collected at the Division of Pharmacology (Otto Loewi Research Center) at the Medical University of Graz with written informed consent. Blood samples were centrifuged at 300 x g and RT for 20min and supernatants were gently mixed with EDTA (2%) in a 1:20 ratio to the plasma volume. After centrifugation at 1000 x g and RT for 15min, the pellet was resuspended in 10 ml wash buffer (containing 140mM NaCl, 10mM NaHCO_3_, 2.5mM KCl, 0.9mM Na_2_HPO_4_ * 2H_2_0, 2.1mM MgCl_2_, 22mM C_6_H_5_Na_3_O_7_, 0.055mM D(+)-Glucose monohydrate and 0.35% BSA) and centrifuged again at 1000 x g and RT for 15min. After another washing step, platelet number was determined using the Sysmex KX-21NTM. Platelets were resuspended in DMEM/F12 (1:1, Gibco, life technologies; Paisley, UK) supplemented with 10% FCS (Gibco), penicillin/streptomycin (Gibco) and L-glutamine (Gibco).

*Co-incubation of platelets with BeWo cells*

For co-incubation with platelets, BeWo cells were seeded in 12-well culture dishes at a density of 2 x 10^5^ cells/well. Next day, culture medium was exchanged with medium containing isolated platelets at a density of 1.5 x 10^8^ platelets/ml in presence or absence of 2-methylthioadenosine diphosphate trisodium salt (Tocris, Bio-techne, Abingdon, UK) in a final concentration of 10 µM. In addition, either forskolin in a final concentration of 20 µM or DMSO as a vehicle control was added. BeWo cells were incubated for indicated durations in a humidified atmosphere of 5% CO_2_ at 37°C.

*Human TGF-β pathway phosphorylation array*

Relative levels of phosphorylation of eight candidate proteins involved in TGF-β signaling were determined in BeWo cells using a membrane based Human TGF-β Pathway Phosphorylation Array (C1 Series, RayBiotech; Norcross, GA, USA). BeWo cell lysates were subjected to membrane arrays according to the manufacturer’s instructions. Chemiluminescent imaging was performed using the FluorChemQ System (Alpha Innotech, Cell Bioscienes; Santa Clara, CA, USA) and signal densities were analysed with AlphaView software version 3.4.0.

**Supplemental tables**

**Supplemental Table 1**

*Immunohistochemistry of platelet marker CD42b in human first trimester placenta*

| **gestational week (mean ± SD)** | **number of cases showing platelets on the surface of villi** | **number of cases showing platelets between ST and VCT layer** | **number of cases showing platelets in intercellular gaps of EVT cell column** |
| --- | --- | --- | --- |
| 5.31 ± 0.43 (n=5) | 4/5 | 2/5 | 3/5 |
| 6.54 ± 0.38 (n=4) | 3/4 | 2/4 | 4/4 |
| 7.17 ± 0.26 (n=5) | 5/5 | 3/5 | 5/5 |
| 8.29 ± 0.29 (n=5) | 5/5 | 2/5 | 3/5* |
| 9.34 ± 0.28 (n=5) | 5/5 | 4/5 | 5/5 |
| 10.39 ± 0.46 (n=4) | 4/4 | 2/4 | 3/4 |
| 11.38 ± 0.54 (n=3) | 3/3 | 0/3 | 1/3* |

Extravillous trophoblast (EVT), syncytiotrophoblast (ST), villous cytotrophoblast (VCT); *one sample did not contain EVT cell column

**Supplemental table 2**

*Human TGF-*β *Pathway Phosphorylation Array*

Data are represented as means ± SD from two independent experiments, values from the DMSO alone experiments were set to 1.

| **Target &**  **Phosphorylation Site** | | **DMSO** | **DMSO + pHPL** | **Forskolin** | **Forskolin + pHPL** |
| --- | --- | --- | --- | --- | --- |
| C-Fos | T232 | 1 ± 0.13 | 1.55 ± 0.40 | 0.92 ± 0.10 | 1.54 ± 0.17 |
| TAK1 | S412 | 1 ± 0.06 | 0.85 ± 0.03 | 1.05 ± 0.11 | 0.96 ± 0.03 |
| SMAD2 | S245/  S250/  S255 | 1 ± 0.20 | 0.57 ± 0.16 | 0.69 ± 0.51 | 0.81 ± 0.38 |
| SMAD4 | T277 | 1 ± 0.04 | 0.76 ± 0.04 | 0.88 ± 0.18 | 0.71 ± 0.10 |
| SMAD5 | S463/  S465 | 1 ± 0.12 | 0.63 ± 0.07 | 0.49 ± 0.03 | 0.61 ± 0.17 |
| ATF2 | T69/  T71 | 1 ± 0.01 | 0.94 ± 0.11 | 0.69 ± 0.02 | 0.58 ± 0.02 |
| c-Jun | S73 | 1 ± 0.17 | 0.63 ± 0.06 | 0.92 ± 0.20 | 0.57 ± 0.18 |
| SMAD1 | S463/  S465 | 1 ± 0.52 | 0.28 ± 0.06 | 0.47 ± 0.03 | 0.41 ± 0.08 |

**Supplemental table 3**

*Antibodies and their working concentrations used for immunostaining and immunoblots*

|  |  |  | IHC | Immunoblot |
| --- | --- | --- | --- | --- |
| ALPPL2 | polyclonal rabbit | PA5-22336,  ThermoFisher Scientific |  | 1:500 |
| CBP | monoclonal rabbit | Clone D6C5, #7389,  Cell signaling |  | 1:1000 |
| CD42b | polyclonal rabbit | 12860-1-AP,  proteintech | 1:1000 |  |
| GAPDH | monoclonal rabbit | Clone 14C10, #2118,  Cell Signaling |  | 1:5000 |
| GCM1 | polyclonal rabbit | P100836_P050,  Aviva Systems Biology |  | 1:250 |
| HLA-G | monoclonal mouse | clone 4H84; BD-Pharmingen | 1:2000 |  |
| P300 | monoclonal rabbit | Clone D8Z4E, #86377,  Cell signaling |  | 1:1000 |
| pCREB(S133) | monoclonal rabbit | Clone 87G3, #9198,  Cell Signaling |  | 1:1000 |
| pSmad2(S465/467) / pSmad3(S423/425) | monoclonal rabbit | Clone D27F4, #8828,  Cell Signaling |  | 1:500 |
| pSmad3(S423/425) | monoclonal rabbit | Clone EP823Y, ab52903, abcam |  | 1:2000 |
| vWF | polyclonal rabbit | F3520,  Sigma-Aldrich | 1:1000 |  |
| Vinculin | polyclonal rabbit | #PA5-29638 |  | 1:1000 |

**Supplemental table 4**

*Primer sequences*

|  | forward | reverse |
| --- | --- | --- |
| ALPPL2 | CCATACCTGGGATTTCCGCCT | CGGTTCCAGAAGTCCGGGTT |
| CDH1 | CAGGATGGCTGAAGGTGACA | ACTGCATTCCCGTTGGATGA |
| CGB | TGAGCCACTCCTGCGCCC | CAGCCCCTGGAACATCTCCA |
| CYC1 | TAGAGTTTGACGATGGCACCC | CCCATGCGTTTTCGATGGTC |
| ERVFRD-1 | ACCGCCATCCTGATTTCCC | GAGGCTGGATAAGCTGTCCC |
| ERVW-1 | CCATGCCGCTGTATGACCAG | GGGTTCCCTTAGAAAGACTCCT |
| EZR | TTGGTTCCGCCCACTCATTC | CCACCTGCACATGGCATCTT |
| GAPDH | ACCCACTCCTCCACCTTTGA | CTGTTGCTGTAGCCAAATTCG |
| GCM1 | TTCCCGGTCACCAACTTCTG | GTAAACTCCCCTGACTTTGTGTT |
| NR4A2 | CGATTTCAGAAGTGCCTGG | TAAACTGTCTGTGCGAACCAC |
| SERPINE1 | GTTCTGCCCAAGTTCTCCCT | ACATGTCGGTCATTCCCAGG |
| YWHAZ | GGTGGCCAATATGGGGATGT | TCCCTTTTATTCCCCGCCAG |

All primers were run at initial 95 °C for 5 min, followed by 40 cycles of 95 °C for 5s and 60 °C for 30s. Melt Curve 60,0°C to 95,0°C: Increment 0,5°C 0:05 Plate Read
